# Supplementary material for: Incident mobility disability, parkinsonism, and mortality in community-dwelling older adults
Source: PLoS One. 2021 Feb 3;16(2):e0246206. doi: 10.1371/journal.pone.0246206 (PMC7857621; doi:10.1371/journal.pone.0246206)
Supplement: S1 Table — (DOCX) [file pone.0246206.s001.docx]

**S1 Table.** A single multi-state Cox model showing the association of vascular risk factors with transitions between different states of motor impairment and death.*

| **State before**  **Transition** | **State after**  **Transition** | **HR (95%CI),**  **p-Value**** |
| --- | --- | --- |
| No Motor impairment | **Mobility disability** | 1.12 (1.02 – 1.24), 0.025 |
| Parkinsonism |  | 0.99 (0.78 – 1.24), 0.865 |
| No Motor impairment | **Parkinsonism** | 1.07 (0.88 – 1.30), 0.482 |
| Mobility disability |  | 0.99 (0.84 – 1.17), 0.881 |
| No motor impairment | **Death** | 1.27 (0.88 – 1.84), 0.208 |
| Mobility disability/No Parkinsonism |  | 1.14 (0.88 – 1.48), 0.326 |
| Parkinsonism/No Mobility disability |  | 1.61 (1.02 – 2.54), 0.042 |
| Mobility disability followed by Parkinsonism |  | 1.18 (0.88 – 1.59), 0.272 |
| Parkinsonism followed by Mobility disability |  | 0.99 (0.74 – 1.32), 0.918 |

* This table summarizes a single multi-state Cox model including 9 transitions among 6 states. Each row examines one of the 9 transitions from an initial state (left column) to a second state (middle column). Each cell in the right column shows hazard ratio (HR), its 95%confidence interval and p value of the association of vascular risk factors with each of the 9 transitions. For additional details see the statistical methods in the text. ^**^ Following Bonferroni adjustment, we assumed p < 0.006 to reject null hypotheses.
