# Supplementary material for: Incident mobility disability, parkinsonism, and mortality in community-dwelling older adults
Source: PLoS One. 2021 Feb 3;16(2):e0246206. doi: 10.1371/journal.pone.0246206 (PMC7857621; doi:10.1371/journal.pone.0246206)
Supplement: S2 Table — (DOCX) [file pone.0246206.s002.docx]

**S2 Table.** A single multi-state Cox model showing the association of vascular diseases with transitions between different states of motor impairment and death.*

| **State before**  **Transition** | **State after**  **Transition** | **HR (95%CI),**  **p-Value**** |
| --- | --- | --- |
| No motor impairment | **Mobility disability** | 1.30 (1.07 – 1.57), 0.007 |
| Parkinsonism |  | 1.09 (0.73 – 1.63), 0.673 |
| No motor impairment | **Parkinsonism** | 1.25 (0.88 – 1.77), 0.215 |
| Mobility disability |  | 1.08 (0.81 – 1.43), 0.618 |
| No motor impairment | **Death** | 1.53 (0.71 – 3.29), 0.278 |
| Mobility disability-no parkinsonism |  | 1.84 (1.26 – 2.70), 0.002 |
| Parkinsonism-no mobility disability |  | 1.53 (0.72 – 3.23), 0.268 |
| Mobility disability, then parkinsonism |  | 1.05 (0.69 – 1.59), 0.825 |
| Parkinsonism, then mobility disability |  | 0.83 (0.46 – 1.51), 0.546 |

^*^ This table summarizes a single multi-state Cox model including 9 transitions among 6 states. Each row examines one of the 9 transitions from an initial state (left column) to a second state (middle column). Each cell in the right column shows hazard ratio (HR), its 95%confidence interval and p value of the association of vascular diseases with each of the 9 transitions. For additional details see the statistical methods in the text. ^**^ Following Bonferroni adjustment, we assumed p < 0.006 to reject null hypotheses.
