# Supplementary material for: Incident mobility disability, parkinsonism, and mortality in community-dwelling older adults
Source: PLoS One. 2021 Feb 3;16(2):e0246206. doi: 10.1371/journal.pone.0246206 (PMC7857621; doi:10.1371/journal.pone.0246206)
Supplement: S3 Table — (DOCX) [file pone.0246206.s003.docx]

**S3 Table.** A single multi-state Cox model showing the association of joint pain with transitions between different states of motor impairment and death.*

| **Motor impairment status before the event** | **Events** | **HR (95%CI), p-Value^*^** |
| --- | --- | --- |
| No motor impairment | **Mobility disability** | 1.34 (1.13 – 1.57), <0.001 |
| Parkinsonism |  | 1.58 (1.08 – 2.32), 0.020 |
| No motor impairment | **Parkinsonism** | 1.04 (0.75 – 1.44), 0.806 |
| Mobility disability |  | 1.28 (0.99 – 1.66), 0.060 |
| No motor impairment | **Death** | 0.79 (0.38 – 1.63), 0.528 |
| Mobility disability-no parkinsonism |  | 1.21 (0.79 – 1.83), 0.380 |
| Parkinsonism-no mobility disability |  | 1.18 (0.50 – 2.77), 0.702 |
| Mobility disability, then parkinsonism |  | 0.83 (0.55 – 1.27), 0.396 |
| Parkinsonism, then mobility disability |  | 0.81 (0.50 – 1.33), 0.410 |

^*^ This table summarizes a single multi-state Cox model including 9 transitions among 6 states. Each row examines one of the 9 transitions from an initial state (left column) to a second state (middle column). Each cell in the right column shows hazard ratio (HR), its 95%confidence interval and p value of the association of any joint pain with each of the 9 transitions. For additional details see the statistical methods in the text. ^**^ Following Bonferroni adjustment, we assumed p < 0.006 to reject null hypotheses.
