# Supplementary material for: Incident mobility disability, parkinsonism, and mortality in community-dwelling older adults
Source: PLoS One. 2021 Feb 3;16(2):e0246206. doi: 10.1371/journal.pone.0246206 (PMC7857621; doi:10.1371/journal.pone.0246206)
Supplement: S5 Table — (DOCX) [file pone.0246206.s005.docx]

**S5 Table.** Primary study findings adjusting for joint pain.

| **Model** | **State before**  **Transition** | **State after**  **Transition** | **HR (95%CI), p-Value** |
| --- | --- | --- | --- |
| **1** | No motor impairment | **Mobility disability** | As the Control. |
|  | Parkinsonism |  | 1.07 (0.88 – 1.31), 0.490 |
| **2** | No motor impairment | **Parkinsonism** | As the Control. |
|  | Mobility disability |  | 3.09 (2.44 – 3.92), <0.001 |
| **3** | Mobility disability followed by parkinsonism | **Death** | As the control. |
|  | Parkinsonism followed by mobility disability |  | 1.21 (0.88 – 1.66), 0.247 |
| **4** | No motor impairment | **Death** | As the control |
|  | Mobility disability/No parkinsonism |  | 1.75 (1.18 – 2.59), 0.005 |
|  | Parkinsonism/No mobility disability |  | 2.87 (1.75 – 4.73), <0.001 |
|  | Mobility disability and parkinsonism |  | 3.86 (2.63 – 5.66), <0.001 |

Each of these 4 models shows the hazard function of one or more of the transitions compared to the hazard of a reference transition controlling for any joint pain. For example, in model 1 we tested if the hazard function of transition from parkinsonism to mobility disability was different from the hazard function of a reference transition from no motor impairment to mobility disability. Additional details are included in the statistical methods in the text.
