# Supplementary material for: Incident mobility disability, parkinsonism, and mortality in community-dwelling older adults
Source: PLoS One. 2021 Feb 3;16(2):e0246206. doi: 10.1371/journal.pone.0246206 (PMC7857621; doi:10.1371/journal.pone.0246206)
Supplement: S6 Table — (DOCX) [file pone.0246206.s006.docx]

**S6 Table.** Primary study findings after exclusion of 14 participants who were diagnosed to have Parkinson disease during the study.

| **Model** | **State before**  **Transition** | **State after**  **Transition** | **HR (95%CI), p-Value** |
| --- | --- | --- | --- |
| **1** | No motor impairment | **Mobility disability** | As the Control. |
|  | Parkinsonism |  | 1.05 (0.85 – 1.29), 0.660 |
| **2** | No motor impairment | **Parkinsonism** | As the Control. |
|  | Mobility disability |  | 3.13 (2.46 – 3.98), <0.001 |
| **3** | Mobility disability followed by parkinsonism | **Death** | As the control. |
|  | Parkinsonism followed by mobility disability |  | 1.18 (0.85 – 1.63), 0.323 |
| **4** | No motor impairment | **Death** | As the control |
|  | Mobility disability/No parkinsonism |  | 1.80 (1.22 – 2.66), 0.003 |
|  | Parkinsonism/No mobility disability |  | 2.93 (1.78 – 4.83), <0.001 |
|  | Mobility disability and parkinsonism |  | 4.06 (2.77 – 5.96), <0.001 |

Each of these 4 models shows the hazard function of one or more of the transitions compared to the hazard of a reference transition after exclusion of 14 participants who were diagnosed to have Parkinson disease during the study. For example, in model 1 we tested if the hazard function of transition from parkinsonism to mobility disability was different from the hazard function of a reference transition from no motor impairment to mobility disability. Additional details are included in the statistical methods in the text.
