# Supplementary material for: Incident mobility disability, parkinsonism, and mortality in community-dwelling older adults
Source: PLoS One. 2021 Feb 3;16(2):e0246206. doi: 10.1371/journal.pone.0246206 (PMC7857621; doi:10.1371/journal.pone.0246206)
Supplement: S7 Table — (DOCX) [file pone.0246206.s007.docx]

**S7 Table.** Primary study findings after replacing parkinsonism with bradykinetic parkinsonism.^*^

| **Model** | **State before**  **Transition** | **State after**  **Transition** | **HR (95%CI), p-Value** |
| --- | --- | --- | --- |
| **1** | No motor impairment | **Mobility disability** | As the Control. |
|  | Bradykinetic parkinsonism |  | 1.10 (0.86 – 1.39), 0.450 |
| **2** | No motor impairment | **Bradykinetic parkinsonism** | As the Control. |
|  | Mobility disability |  | 3.29 (2.49 – 4.35), <0.001 |
| **3** | Mobility disability followed by bradykinetic parkinsonism | **Death** | As the control. |
|  | Bradykinetic parkinsonism followed by mobility disability |  | 1.23 (0.86 – 1.76), 0.259 |
| **4** | No motor impairment | **Death** | As the control |
|  | Mobility disability/No bradykinetic parkinsonism |  | 1.74 (1.23 – 2.48), 0.002 |
|  | Bradykinetic parkinsonism/No mobility disability |  | 2.24 (1.27 – 3.95), 0.005 |
|  | Mobility disability and bradykinetic parkinsonism |  | 3.45 (2.40 – 4.95), <0.001 |

^*^Bradykinetic parkinsonism was defined as presence of bradykinesia and at least one other parkinsonian sign (which are rigidity, tremor, parkinsonian gait).

Each of these 4 models shows the hazard function of one or more of the transitions compared to the hazard of a reference transition after replacing parkinsonism with bradykinetic parkinsonism. For example, in model 1 we tested if the hazard function of transition from bradykinetic parkinsonism to mobility disability was different from the hazard function of a reference transition from no motor impairment to mobility disability. Additional details are included in the statistical methods in the text.
