# Supplementary figures and images for: Incident mobility disability, parkinsonism, and mortality in community-dwelling older adults
Source: PLoS One. 2021 Feb 3;16(2):e0246206. doi: 10.1371/journal.pone.0246206 (PMC7857621; doi:10.1371/journal.pone.0246206)

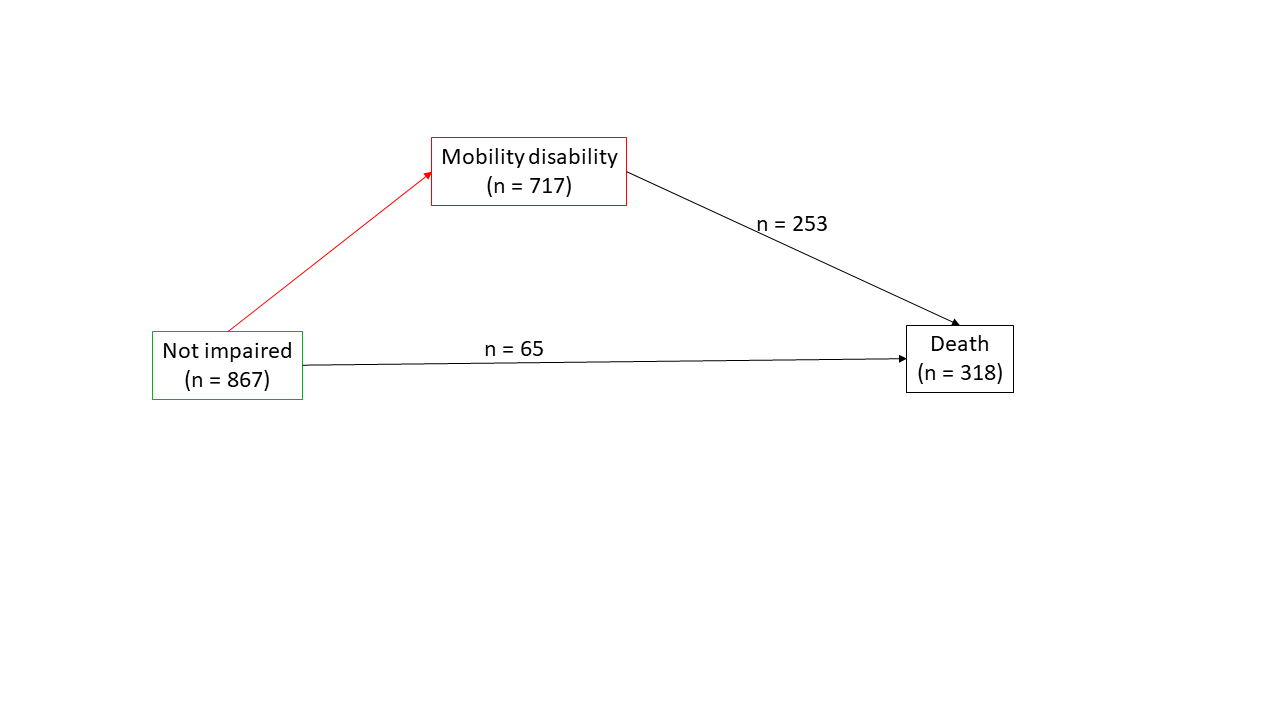

Supplement: S1 Fig — This figure describes a multi-state model of incident mobility disability and death. The boxes show the 3 possible states and the arrows show the 3 possible transitions. (TIF) [file pone.0246206.s008.tif]

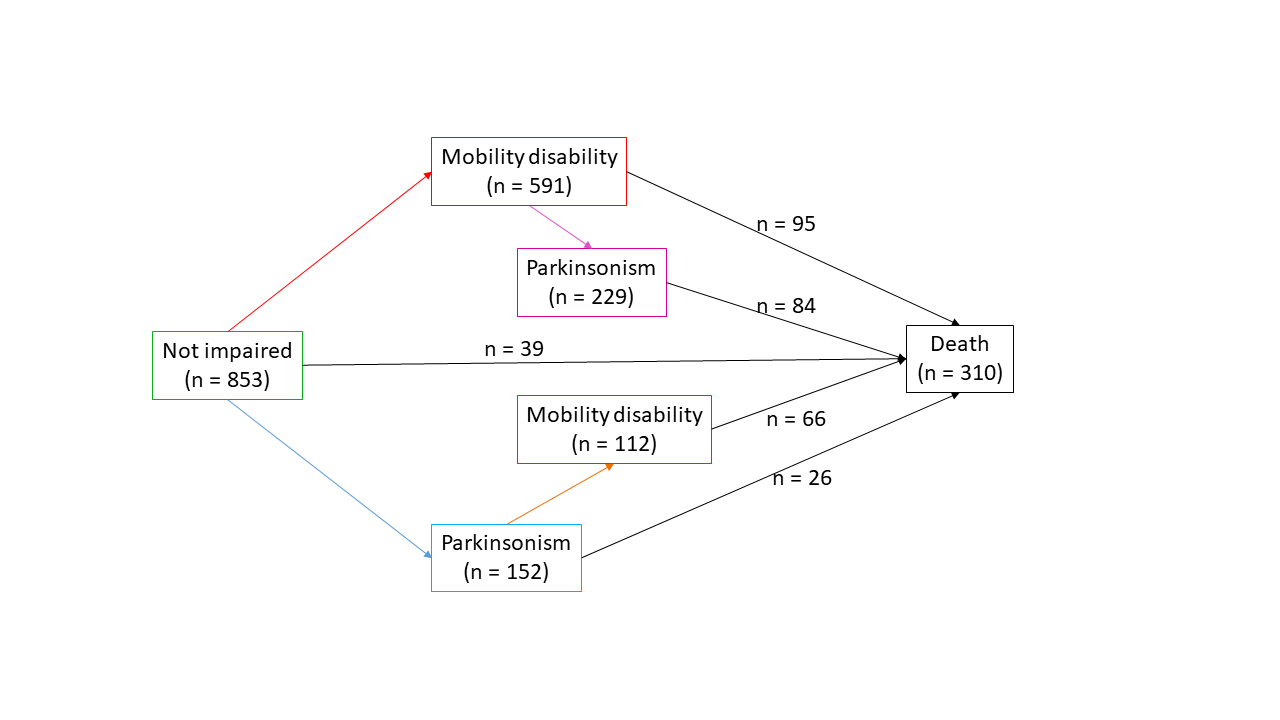

Supplement: S2 Fig — This figure illustrates the frequencies of participants in each of the six states and nine transitions examined in a multi-state model of incident mobility disability, bradykinetic parkinsonism, and death. Mobility disability was defined as gait speed less than 0.55 m/s in an 8-feet walk test. Parkinsonism was defined by presence of at least two of the four parkinsonian signs (bradykinesia, rigidity, tremor, parkinsonian gait). All participants included in this study were initially without mobility disability or parkinsonism at the analytic baseline. During the course of this study, nine paths for transition were possible between the baseline state of no motor impairment, four intermediate states of varying degrees of motor impairment, and the final absorbing state of death. (TIF) [file pone.0246206.s009.tif]

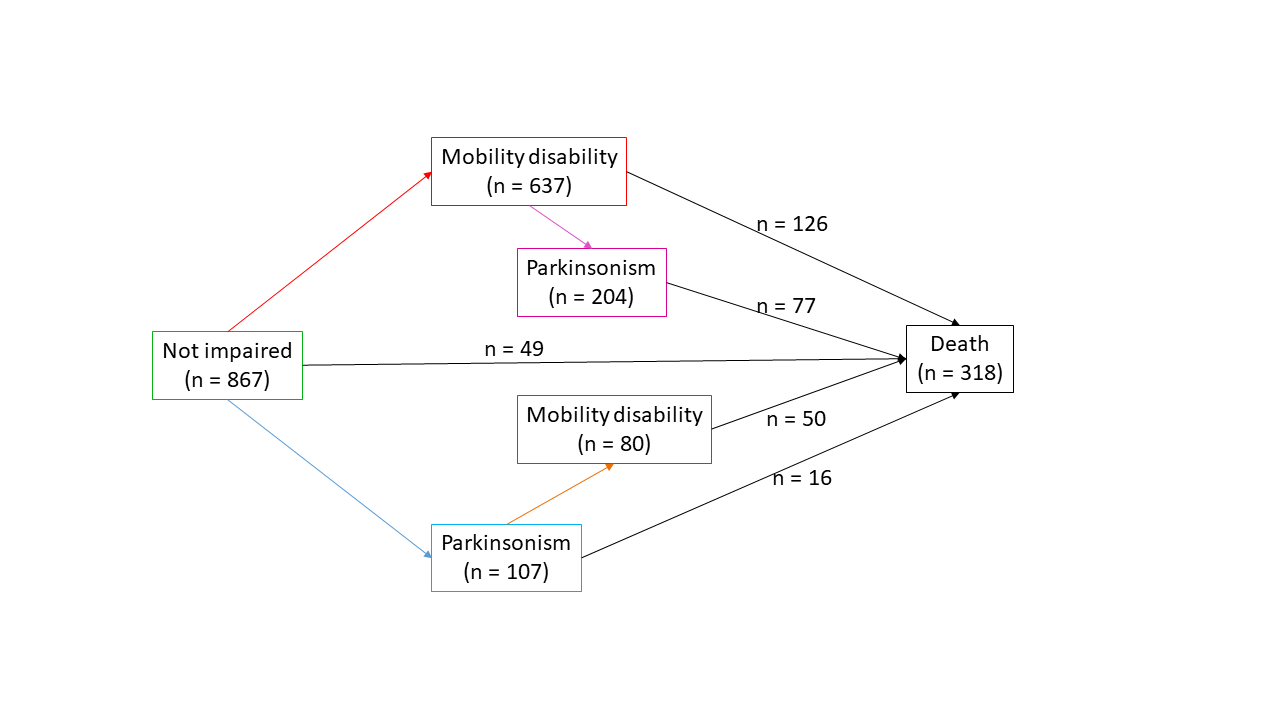

Supplement: S3 Fig — This figure illustrates the frequencies of participants in each of the six states and nine transitions examined in a multi-state model of incident mobility disability, bradykinetic parkinsonism, and death. Mobility disability was defined as gait speed less than 0.55 m/s in an 8-feet walk test. Bradykinetic parkinsonism was defined as presence of bradykinesia and at least one other parkinsonian sign (which are rigidity, tremor, parkinsonian gait). All participants included in this study were initially without mobility disability or parkinsonism at the analytic baseline. During the course of this study, nine paths for transition were possible between the baseline state of no motor impairment, four intermediate states of varying degrees of motor impairment, and the final absorbing state of death. (TIF) [file pone.0246206.s010.tif]
